# New Promises and Challenges on Inflammation and Atherosclerosis: Insights From CANTOS and CIRT Trials

**DOI:** 10.3389/fcvm.2019.00090

**Published:** 2019-07-02

**Authors:** Raymond D. Palmer, Mauro Vaccarezza

**Affiliations:** ^1^Helium-3 Biotech, South Perth, WA, Australia; ^2^Faculty of Health Sciences, School of Pharmacy and Biomedical Science, Curtin University, Perth, WA, Australia

**Keywords:** cardiovascular risk, acute coronary syndrome, atherosclerosis, ischemic heart disease, vascular disease

Atherosclerosis is still a great burden on human health and scientific achievements of the past 30 years have definitively proven the inflammatory causes of the disease (1, 2) highlighted by the results of the CANTOS trial (3). Canakinumab inhibition of interleukin-1 (IL1)-beta has been demonstrated to provide protection against cardiovascular (CV) risk irrespective of lipid levels in a cohort of patients with high C–reactive protein (CRP) levels despite achieving blood lipid control, as measured by high sensitivity C-Reactive Protein (hsCRP) (3).

The final demonstration of atherosclerosis as an inflammatory-driven pathology is driving research toward new anti-inflammatory approaches that could influence the natural history of the disease and change the clinical paradigms of therapy. Considering the costs of canakinumab and exploiting different avenues of anti-inflammatory therapies, a more affordable approach has been proposed by the authors of the CANTOS study (4). Low dose methotrexate (MTX) proposed in the CIRT study (5) could be an alternative approach aimed to lower the inflammatory burden and to deliver a beneficial effect on patient CV risk (4). Data from this study is now available (5) and demonstrates the feasibility of the low MTX treatment and at the same time a somewhat surprising lack of effect on CV risk.

In this published randomized trial low dose MTX did not reduce IL1-beta levels or hsCRP and did not have any meaningful effect on cardiovascular disease (CVD) risk (a slight increase in skin tumors was registered over time in the MTX treated population).

These results, while disappointing, are not entirely unexpected: MTX activity and the molecular pathways involved are different from the canakinumab mechanism(s) of action, targeting less relevant pathogenic signal cascades in atherosclerosis. Of note the CIRT trial was to a large extent based on the data from patients affected by Rheumatoid Arthritis (RA) on low dose MTX in whom (as observed) was shown to decrease both CV risk and inflammatory cytokine levels (4, 6–8). The genesis of atherosclerotic lesions in RA remains an open and unsolved line of enquiry for further research: Is atheromata driven by the same mechanism as classical atherosclerosis or is there a disparate molecular pathway hidden among the CV framework? Maybe a greater extent of T cell involvement could be a possible cause but only further research and observation will deliver the dataset required. There is indeed mounting evidence that T cell subpopulations are involved in the pathogenesis of atherosclerosis (9). Thus, in principle, we should be cautious in comparing the results of the two trials without considering the differences in pathways and biological response.

This caveat notwithstanding, what are the hallmarks that we could extrapolate from this nevertheless very interesting study?

The first consideration is related to the target population of each study (CANTOS and CIRT): as highlighted by the authors (5) both trials involved heavily pre-treated subjects and prior randomization, but the two populations used for CANTOS and the CIRT study were actually different and not superimposable.

CANTOS included patients after myocardial infarction (MI) with residual inflammatory risk as measured by hsCRP levels higher than 2 mg/L despite aggressive therapy. CIRT had patients with indirect evidence of inflammatory risk (not defined by hsCRP levels) determined by the presence of diabetes or metabolic syndrome (pathologies associated with a pro-inflammatory *pabulum*). Levels of hsCRP were 4.2 mg/L as a median baseline in CANTOS, whereas the median hsCRP level was 1.6 mg/L at randomization in the CIRT population (3, 5).

Thus, in the CANTOS trial the pro-inflammatory *pabulum* was definitely more pronounced (as *per* hsCRP levels) and this situation could in part explain the different results of the two studies.

Although both trials studied a high risk population, the event rates for the same primary endpoint, a composite of non-fatal MI, non-fatal stroke and CV death were considerably higher in CANTOS (4.5/100 person years) compared with CIRT (3.43/100 person years). As an interesting point of fact, CIRT event rates were at the level of those patients achieving an hsCRP < 2mg/L with canakinumab in CANTOS (3.28/100 person years). Median hsCRP levels in CIRT were indeed lower than in the CANTOS placebo arm (as reported above).

Regarding MTX mechanisms of action, it has been reported that MTX decreases elevated CV risk in patients with RA, along with suppression of elevated CRP levels [CRP being less sensitive and precise than hsCRP; (6, 7)]. So, it seems that the inflammatory burden in the overall CIRT study population may not have exceeded a certain threshold to benefit from anti-inflammatory therapies, thus a positive role of MTX in vascular wall disease is not entirely ruled out (6–8). These assumptions are highlighted by the fact that we do not really know the exact mechanism of action with low dose MTX (10): the most stringent experimental proof is an increase in adenosine (an important anti-inflammatory mediator) and adenosine-related metabolite levels in treated patients (10) and other possible mechanisms of action are proposed (10). At this time, it is not known if the proposed mechanism(s) of action of low dose MTX are relevant or scarcely relevant to the pathogenesis of atherosclerosis or relevant only in some stages of the disease. More studies focusing on the role of T lymphocytes and adaptive immunity (9) in the natural history and pathogenesis of atherosclerosis are warranted to better understand the molecular action(s) and the eventual role of low dose MTX.

A secondary and important aspect is that targeted inhibition of inflammatory pathways is warranted in the case of atherosclerosis for a significant therapeutic effect.

It is worth noting that in the CANTOS trial a definitive reduction in Interleukin-6 (IL6) and hsCRP levels (known inflammatory biomarkers downstream to IL1-beta signaling pathway) were substantial, whereas no changes were registered in the CIRT study.

It is conceivable that robust reduction of CV risk is dependent on the specific pathways targeted. Of note, recent experimental results on the involvement of the NLRP3 pathway (a main trigger of IL1-beta release) seems to confirm the pivotal role of specific signaling pro-inflammatory routes in atherosclerosis pathophysiology (11–13). NLRP3 pathway inhibition could indeed effectively target inflammatory pathways in atherosclerosis (11–13). In this scenario it is interesting to report the recent Novartis withdrawal of EU filing for approval of canakinumab for CV risk reduction; the FDA had rejected their application for the US market as well^1^. According to the available reports the regulatory agencies were still concerned that the beneficial effects might not outweigh the increased risk of serious infections [see also (14–16)]. This is noteworthy since canakinumab did reduce the overall CV mortality rate by about 30% in patients that responded with a reduction in hsCRP and IL6 after the first injection. “Non-responders” could conveniently be identified by one follow-up hsCRP measurement at 3 months and treatment could be terminated in these patients sparing them the risk of experiencing serious side effects. In “responders (hsCRP < 2 mg/L)” the benefits obviously outweigh the risks resulting in an astonishing NNT (Number–Needed to Treat) of 16! Based on this data we are of the opinion that the research community needs to scrutinize the decisions being made by the regulatory authorities (under exclusion of the public) across the scientific literature; as this research sparks further critical discussions, so too will other reviews cause different interpretations of the data. Other specialists in the field of Internal Medicine (such as rheumatologists) are used to suppress the immune system to alleviate rheumatic and autoimmune disease burden while balancing the risk for infections. There is no reason to believe that cardiologists would not be able to make comparable judgements based on that data. Thus, the risk of serious adverse infectious effects related to canakinumab use is clear, but we think that it could be manageable with careful patient selection (i.e., excluding in future studies patients with diabetes mellitus), and with a multi-specialist approach to CV patients with a residual inflammatory “pabulum.” New approaches (such as the NLRP3 inhibition mentioned above) may be a possible solution in delivering therapeutic specificity and efficacy while protecting patients from serious adverse side effects.

In conclusion, we have to be grateful for the authors of the CANTOS and CIRT studies who both delivered important information to the medical community in regards to the pathogenic mechanisms of atherosclerosis and its potential clinical translations. Understanding differences and caveats in the CANTOS, CIRT, and other studies involving additional anti-inflammatory therapies for CVD (14–20) is likely to be useful in designing future therapeutics for atherosclerosis. Critically, more studies are needed and it is not yet time for a robust clinical application of these findings, but the CANTOS and CIRT trials have paved the way for new personalized and promising avenues for controlling this still devastating disease.

## Author Contributions

RP: literature review, an advanced draft of the manuscript, final editing, review, and approval of the manuscript. MV: idealization and scientific input, literature review, an initial draft of the manuscript, final review, and approval of the manuscript.

### Conflict of Interest Statement

RP is Chief Scientific Officer of Helium-3 Biotech. The remaining author declares that the research was conducted in the absence of any commercial or financial relationships that could be construed as a potential conflict of interest.

## Footnotes

^1^http://www.pmlive.com/pharma_news/novartis_withdraws_eu_filing_for_canakinumab_1272895 (accessed April 2, 2019); https://www.epgonline.org/global/news/novartis-withdraws-from-ema-its-application-for-canakinumab-for-the-prevention-of-serious-cv-events-.html (accessed April 2, 2019); https://endpts.com/fda-slaps-down-novartis-blockbuster-pitch-for-canakinumab-so-what-went-wrong/ (accessed April 2, 2019); https://business.medicaldialogues.in/fda-snubs-novartis-bid-to-repurpose-inflammation-drug-canakinumab-for-heart-attacks/ (accessed April 2, 2019).
